# Biased Signaling and Its Role in the Genesis of Short- and Long-Acting β_2_‑Adrenoceptor Agonists

**DOI:** 10.1021/acs.biochem.5c00148

**Published:** 2025-08-07

**Authors:** Ngan T. N. Phan, Leire Borrega-Roman, Bradley L. Hoare, Clare R. Harwood, Natalie Geary, Wolfgang Guba, Yongqi Han, Vladimirs Zenko, Eline J. Koers, Arne C. Rufer, Uwe Grether, Dmitry B. Veprintsev, David A. Sykes

**Affiliations:** † Division of Physiology, Pharmacology & Neuroscience, School of Life Sciences, 6123 University of Nottingham, Nottingham NG7 2UH, U.K.; ‡ Centre of Membrane Proteins and Receptors (COMPARE), University of Nottingham, Midlands, Nottingham NG7 2UH, U.K.; § Z7 Biotech Limited, 1 Poplars Court, Lenton Lane, Nottingham NG7 2RR, U.K.; ∥ Roche Pharma Research & Early Development, Therapeutic Modalities, Roche Innovation Center Basel, F. Hoffmann-La Roche Ltd., 4070 Basel, Switzerland

## Abstract

Drug discovery is a costly and time-intensive process that is often limited by efficacy issues and unforeseen side effects. GPCR-targeting ligands, which account for one-third of marketed drugs, have been shown to exhibit biased signaling and preferential activation of one signaling pathway over another. While designing biased ligands is a recent advancement, their therapeutic benefits remain uncertain. However, the success of existing drugs raises the following question: do they inherently exhibit signaling bias that enhances efficacy or safety? This study examines the signaling profiles of short- and long-acting β_2_AR agonists (SABAs and LABAs), key treatments for asthma and COPD, using biosensors to measure G protein and β-arrestin coupling. Older SABAs, such as isoprenaline and isoetharine, show minor G protein bias, while newer agents, such as salbutamol, demonstrate significant G protein bias. Among LABAs, salmeterol shows greater G protein bias compared to that of the more balanced formoterol. This shift toward G protein bias over 50 years reflects efforts to improve asthma treatments. The increased bias results from reduced ligand–receptor residence times and weaker receptor−β-arrestin complex formation, contributing to the enhanced efficacy. Despite the potential advantages, a systematic evaluation of signaling bias remains underutilized in drug development. Early-stage, high-throughput tools to assess signaling profiles could improve candidate selection, reduce late-stage failures, and minimize side effects. We advocate for the routine integration of biosensors for quantifying signaling bias, optimizing compound selection, and enhancing therapeutic outcomes.

## Graphical abstract

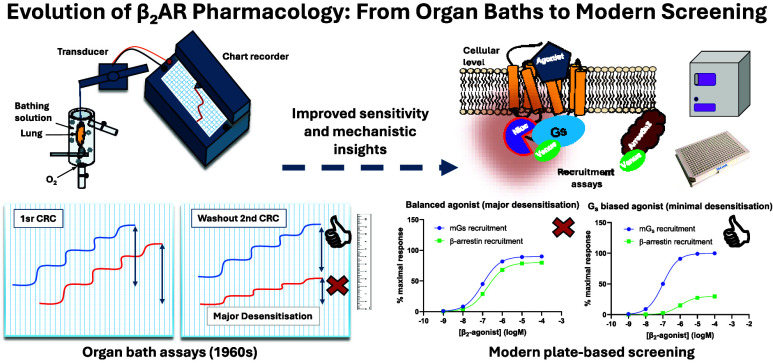

## Introduction

One of the major challenges in GPCR drug discovery is the high attrition rate during late-stage development, including preclinical testing and clinical trials, primarily due to inadequate efficacy and unexpected side effects.^1–3^ The ability to predict drug–receptor side effects relies heavily on our extensive knowledge of cellular signaling pathways and reported clinical outcomes.^4^ To improve the current paradigm and increase the likelihood of success, more advanced and predictive tools are needed to assess the pharmacological properties of the drug candidates. Such tools would allow for a deeper understanding of drug function at the molecular and cellular levels earlier in the discovery pipeline, helping to identify potential safety and efficacy issues before they arise in later stages.

Increasing a drug’s therapeutic potential can be achieved usually in one of two ways. For antagonists, we can improve their selectivity for the therapeutic target and, in doing so, lessen off-target effects. For agonists, in addition to improving receptor selectivity, we can attempt to generate a unique receptor activation profile, one which permits the recruitment of therapeutic effector proteins at the expense of those causing side effects, so-called signaling bias or functional selectivity.^5,6^

The concept of signaling bias and its potential for therapeutic application evolved from the observation that receptors could couple to more than one signaling transduction pathway, and was first described by Roth and Chuang.^7,8^ For agonists that demonstrate signaling bias, it has been suggested that distinct active conformations must play a role in the generation of differential signals.^9–11^ This stems from the idea that GPCR function and drug–receptor interactions cannot be simply described by bimodal “on–off” switches, reflecting activation by agonist and inactivation by antagonists.^12^ No longer do we think of GPCRs as existing in two functionally relevant states— inactive (R) and active (R*)—but as highly dynamic membrane-bound proteins with multiple active conformational states (R*^n^),^13^ which lead to activation of different cellular signaling pathways, so-called ‘pluridimensional efficacy.’^14^

The detection of more selective or biased ligands for GPCRs is hampered by a lack of high-throughput signaling (HTS) techniques, capable of revealing subtle differences in receptor bias toward a particular effector protein following receptor activation. Two relevant approaches have been successfully applied to GPCRs to capture these active-like states; the first involves the use of a single-chain camelid antibody (nanobodies) to stabilize the active state of a GPCR.^15^ Application of such a nanobody mimics the cooperative effects of the heterotrimeric G protein or other effectors such as β-arrestin. An alternative methodology has been provided through the realization that only the GTPase domain of the Gα subunit makes a significant contact with the active β_2_AR. As a consequence, the GTPase domain of the Gα subunit has been engineered and thermostabilized to generate a mini-G protein, mini-G_s_ (mG_s_).^16^ The main advantage of the mG protein is that it can form a high-affinity state with any GPCR that binds this heterotrimeric G protein.^17,18^ A similar approach has been successfully engineered to study β-arrestin binding,^19^ facilitating conformational screening on the G protein and arrestin-coupled forms of a receptor.

While the intentional design of biased ligands is a recent innovation,^20^ many marketed drugs may exhibit functional bias as an unintended result of optimization during development. Drug discovery inherently favors compounds with desirable clinical outcomes, raising the question of whether existing drugs possess inherent signaling bias that enhances their efficacy or safety.

The β_2_AR remains one of the most well-studied and widely targeted GPCRs, with a longstanding role in the treatment of respiratory diseases such as asthma and COPD.^21^ In this study, we hypothesized that the clinical use of existing β_2_AR ligands, including short-acting β_2_ agonists (SABAs) and long-acting β_2_ agonists (LABAs), would reflect their observed pharmacology, driven by improvements in efficacy and signaling bias.^22,23^ This study aims to investigate the signaling bias of clinically used β_2_AR agonists (SABAs and LABAs) using existing biophysical screening methods with the goal of uncovering functional selectivity that may contribute to their efficacy and safety profiles, thereby providing valuable insights into their clinical performance and guiding future drug discovery strategies. The simple nanoBRET-based screening methods employed are fully capable of distinguishing between β-arrestin-biased and G protein-biased ligands.^18,24^ These methods assess both efficacy and potency independently of signal amplification, which is a confounding factor in the measurement of relative ligand signaling efficacy and bias.^25,26^

Our detailed pharmacological analysis of β_2_AR agonist G protein and arrestin signaling suggests that the search for clinical efficacy has driven functional selectivity and reveals key factors contributing to the signaling bias of drugs developed over the past 50 years, when profiling for sustained efficacy was primarily conducted using organ bath experiments with washout protocols. Notably, our findings highlight the role of agonist–receptor complex binding kinetics in dictating arrestin recruitment and its potential impact on the side effect profiles of clinically used β_2_AR-agonists.

## Materials and Methods

### Materials

The T-REx-293 cell line was obtained from Invitrogen (CA, U.S.A). T75 and T175 mammalian cell culture flasks were purchased from Fisher Scientific (Loughborough, U.K.). All cell culture reagents, including phosphate-buffered saline (PBS) and fetal calf serum (FCS), were purchased from Sigma-Aldrich (Gillingham, U.K.), except for blasticidin and zeocin, which were obtained from Gibco (MA, U.S.A). Polyethylenimine (PEI) (25 kDa) was obtained from Polysciences Inc. (PA, U.S.A), and culture plates were from Greiner Bio-One (code 655098 Kremsmünster, Austria). Hanks′ balanced salt solution (H8264), HEPES (4-(2-hydroxyethyl)-1-piperazineethanesulfonic acid), bovine serum albumin (BSA) heat shock fraction, protease-free, fatty acid-free, essentially globulin-free (A7030), and poly-d-lysine were obtained from Sigma-Aldrich (Gillingham, U.K.).

Salmeterol xinafoate was obtained from Clinisciences, Limited. Formoterol hemifumarate was obtained from APExBIO (TX, U.S.A). Salbutamol hemisulfate, ICI118551 hydrochloride, Isoetharine mesylate salt, and isoprenaline hydrochloride were purchased from Sigma-Aldrich (Gillingham, U.K.). Alprenolol (HY-B1517) was purchased from MedchemExpress, Limited. Nano-Glo luciferase substrate was obtained from Promega (WI, U.S.A). All other chemicals were purchased from Sigma-Aldrich (Gillingham, U.K.).

### mG_s_ Protein and β-Arrestin Recruitment Assays

T-REx-293-β_2_AR-nLuc (ADRB2_HUMAN, UniProtKB P07550) cells stably expressing fluorescently labeled mG_s_ protein and β-arrestin2 were used to assess effector recruitment. Cultured cells were harvested upon reaching 70% confluency and plated at a seeding density of 50,000 cells per well in poly-D coated 96-well view plates. The cells were grown for 48 h until they reached confluency and were then stimulated with 1 μg/mL tetracycline for a further 48 h to induce receptor expression. Cell culture media was then aspirated from the wells, and the cells were washed with assay buffer, 100 μL/well Hanks′ balanced salt solution (HBSS) containing 0.1% BSA and 5 mM HEPES. Following the wash, assay buffer containing 10 μM furimazine, 90 μL/well, was added to the wells. The plate was then incubated at 37 °C for 15 min to allow the nLuc substrate furimazine to fully equilibrate and then transferred to the Pherastar FSX preset to 37 °C. Three BRET cycles of 1 min intervals were performed to assess basal nanoBRET levels, following which 10 μL of each compound, diluted in assay buffer, was added to the assay plate, which was read at 1 min intervals for 30 min.

Compounds were serially diluted in DMSO in polypropylene plates (100× final concentration) and then transferred to a second 96-well dilution containing assay buffer (10× final concentration and 10% DMSO). Finally, the compounds were transferred to the assay plates. 1% DMSO in assay buffer served as the vehicle control, and the positive control for β_2_AR experiments was formoterol (1 μM).

In a series of separate experiments conducted at room temperature to examine the reversibility of recruitment responses, an EC_80_ concentration of each agonist was applied to cells. Once responses reached their peak (approximately 20 min), a high concentration of ICI118551 (10 μM) was added to initiate reversal. Responses were subsequently monitored for up to 90 min for mG_s_ and up to 60 min for β-arrestin2.

### Concentration Response Curves

mG_s_ responses were taken at 30 min (peak), while β-arrestin2 responses were taken at their peak response time. A typical time course in the mG_s_ and β-arrestin2 assays for the agonist isoprenaline is shown in Supporting Information Figure S1A and B. mG_s_ and β-arrestin2 concentration response data were fitted to sigmoidal (variable slope) curves using a “four-parameter logistic equation”:

1 $$Y=\mathrm{Bottom}+\frac{\left(\mathrm{Top}-\mathrm{Bottom}\right)}{1+10^{\left(\log{\mathrm{EC}}_{50}-X\right)\times \mathrm{Hillslope}}}$$

where Bottom and Top are the lower and upper plateaus of the agonist concentration response curves. LogEC_50_ is the concentration of agonist that gives a half-maximal effect, and Hillslope is the unitless slope factor or Hillslope.

In addition to measuring peak mG_s_ and β-arrestin2 responses, we performed an area under the curve (AUC) analysis to capture recruitment over time. Baseline (vehicle) responses were subtracted from those elicited by each agonist concentration prior to the AUC calculation. Total AUC values were then computed in Prism and fitted to sigmoidal (variable slope) curves using the four-parameter logistic equation as described above.

### Bias Factor Calculations

To determine the relative effectiveness of the compounds to activate the different signaling pathways, the difference between the log­(*E*_max_/EC_50_) values was calculated. Analysis was performed as described by previously^27,28^ to determine Δlog­(*E*_max_/EC_50_):

2 $$\Delta \log\left(\frac{E_{\max}}{{EC}_{50}}\right)=\log\left(\frac{E_{\max}L1}{{EC}_{50}L1}\right)-\log\left(\frac{E_{\max}L2}{{EC}_{50}L2}\right)$$

where ligand 2 (*L*2) is the reference compound and ligand 1 (*L*1) is the test compound. Δlog­(*E*_max_/EC_50_) values were determined using formoterol as the reference agonist.

Using the estimates of agonist activity Δlog­(*E*_max_/EC_50_) for the test and reference agonists, pathway log bias was calculated as follows:

3 $$\Delta \Delta \log\left(\frac{E_{\max}}{{EC}_{50}}\right)=\Delta \log\left(\frac{E_{\max}}{{EC}_{50}}\right)P1-\Delta \log\left(\frac{E_{\max}}{{EC}_{50}}\right)P2$$

where pathway 1 (*P*1) is mG_s_-dependent recruitment and pathway 2 (*P*2) is β-arrestin2 recruitment. Bias factors were calculated by taking the antilog of the ΔΔlog­(*E*_max_/EC_50_).

### Estimation of Pathway Error

Error on the pathway bias was calculated using the following formula:

4 $${\mathrm{SEM}}_{\Delta \Delta \log\left(\frac{E_{\max}}{{\mathrm{EC}}_{50}}\right)}=\sqrt{{\mathrm{SEM}}_{\Delta {\log\left(\frac{E_{\max}}{{\mathrm{EC}}_{50}}\right)}_{P1}}^{2}+{\mathrm{SEM}}_{\Delta {\log\left(\frac{E_{\max}}{{\mathrm{EC}}_{50}}\right)}_{P2}}^{2}}$$

This aligns with the results of error propagation derivation outlined in van der Westhuizen et al.,^23^ which justifies the use of the Gaussian propagation law. A full derivation of the above equation and its justification can be found in the Supporting Information.

### Dissociation from Receptor–Effector Complexes

mG_s_ and β-arrestin2 response data were fitted to a one-phase exponential decay equation to derive the dissociation 1/2 life (0.693/*k*_off_) of each ligand:

5 $$Y=\left(Y_{0}-\mathrm{Plateau}\right)\times \exp^{\left(-k_{\mathrm{off}}\times X\right)}+\mathrm{Plateau}$$

where

*X* = time (min), *Y* = total binding (BRET units), *Y*_0_ = *Y* at time 0, Plateau = binding at very long times in units of *Y*, and *k*_off_ = dissociation rate constant (min^–1^).

### Signal Detection and Data Analysis

Signal detection was performed on a BMG PHERAstar FSX plate reader (BMG Labtech, Offenburg, Germany) fitted with a BRET1 plus (emA. 475/30 nm, emB. 535/30 nm) optic module and MARS software purchased from BMG Labtech (Offenburg, Germany). GraphPad Prism 9.2 was purchased from GraphPad Software (San Diego, U.S.A.). Microsoft Excel XP was purchased from Microsoft (Washington, DC, U.S.A.). Correlations were determined using Pearson’s analysis, and the resulting relationships were represented using Deming’s analysis.

## Results

### nanoBRET-Based β_2_AR mG_s_ and β-Arrestin2 Recruitment Assays Enable Reliable Determination of Signaling Bias

The principle of these assays is based on proximity between the nLuc enzyme, which is genetically incorporated at the C-terminal tail of the GPCR, and a Venus fused effector protein, which is recruited to the C-terminal tail of the receptor on agonist binding (see Figure 1). Pathway bias occurs when either the effector has an apparent higher affinity for the ligand-bound GPCR, as denoted by a difference in *E*_max_ resulting in an increase in maximal effector binding (or % control response), or the ligand recruits the effector protein more efficiently, reflected in EC_50_ or potency changes.

**Figure 1:**
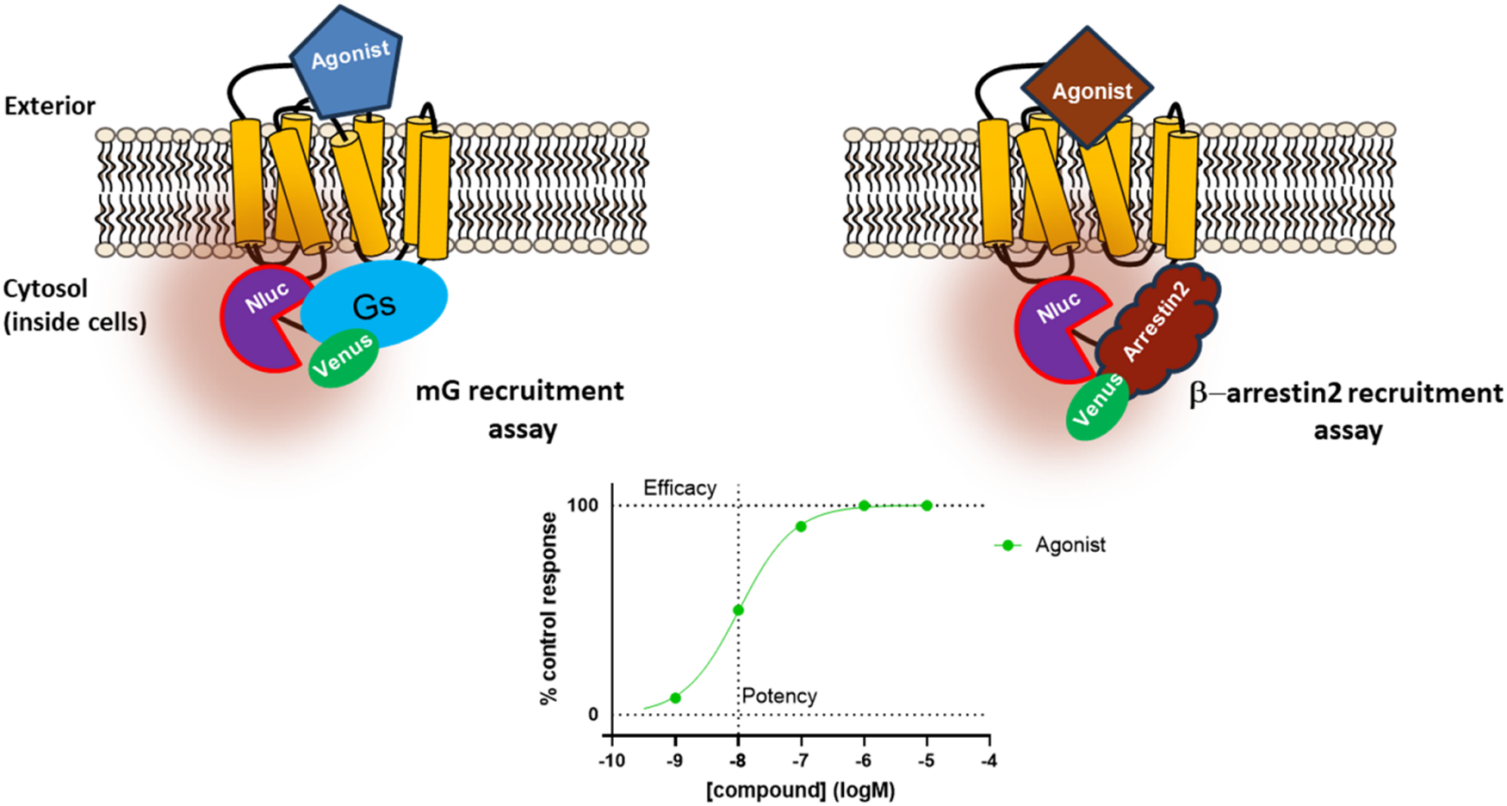
mG_s_ and β-arrestin2 recruitment assays. The principle of these assays is based on proximity between the nLuc enzyme, which is attached to the C-terminal tail of the GPCR, and the Venus fused effector protein, which is recruited to the C-terminal tail on agonist binding to the receptor. Bias occurs when the effector has either an apparent higher affinity for the ligand-bound GPCR, as denoted by a difference in *E*_max_ resulting in an increase in maximal effector binding (or % control response), or the ligand recruits the effector protein more efficiently, reflected in EC_50_ or potency changes.

The β_2_AR agonist profiled in these nanoBRET-based effector recruitment assays produced concentration-dependent increases in the recruitment of both mG_s_ and the β-arrestin2 protein. The BRET ratios obtained were normalized using formoterol as the reference agonist (100% response). Notably, three of the four SABAs profiled isoprenaline, isoetharine, and salbutamol showed significant bias toward mG_s_, at least in terms of potency (see Figure 2A–C). The fourth, fenoterol, was only marginally biased toward mG_s_ in terms of its potency (Figure 2D). In addition to the four SABAs profiled, two LABAs were also studied. While formoterol appears to possess a more balanced profile, salmeterol showed bias toward the mG pathway at least in terms of observed *E*_max_ (see Figure 2E and F). Tulobuterol, which is administered transdermal and can be considered a LABA, was also profiled and appears to be unique, in that it recruits mG_s_ very weakly, but also shows no effect on β-arrestin2 recruitment (see Figure 2G). Finally, the response to alprenolol is shown; this molecule is usually only seen as an agonist of G protein recruitment under conditions where signal amplification is prevalent (see Figure 2H), highlighting the great sensitivity of the systems under study. In general, the errors associated with ligand pathway *E*_max_ and pEC_50_ values were low, highlighting the reproducibility of the measurements and the suitability of this technology for high-throughput profiling in drug discovery (see Table 1).

**Figure 2:**
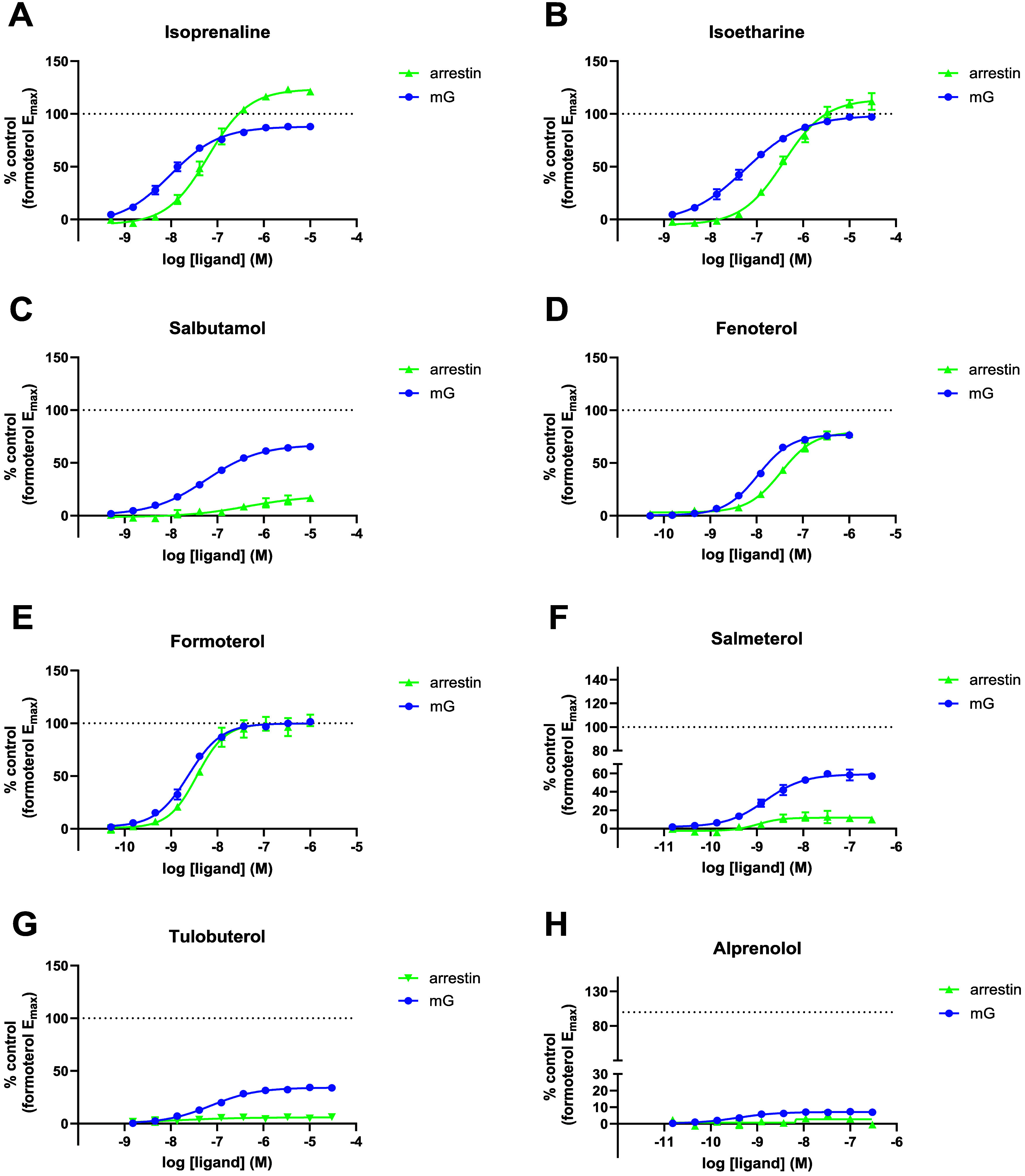
BRET-based β_2_AR mG_s_ and β-arrestin2 recruitment assays. β_2_AR mG_s_ and β-arrestin2 concentration response curves for the β_2_AR agonists (A) isoprenaline, (B) isoetharine, (C) salbutamol, (D) fenoterol, (E) formoterol, (F) salmeterol, (G) tulobuterol, and (H) alprenolol. The BRET ratios obtained were normalized using the pathway balanced ligand formoterol as the reference agonist (100% response). Data shown are the mean ± SEM from 3 or more experiments, with mG_s_ and β-arrestin2 responses to each agonist conducted on the same plate.

**Table 1:** BRET-Based mG_s_ and β-Arrestin2 Recruitment Maximal Effect (*E*_max_) and Potency (pEC_50_) Data Plus Bias Factors^a^

β_2_AR					
	mG_s_		β-arrestin2		
compound	pEC_50_	*E*_max_	pEC_50_	*E*_max_	log bias factor (mG_s_/Arr2)
Formoterol	8.65 ± 0.08 (4)	100 ± 2.9 (4)	8.44 ± 0.03 (3)	100 ± 6.8 (3)	0 ± 0.10
Isoprenaline	7.99 ± 0.08 (8)	87.1 ± 1.9 (8)	7.17 ± 0.10 (7)	122.3 ± 0.9 (7)	0.48 ± 0.14
Isoetharine	7.21 ± 0.09 (4)	97.8 ± 1.4 (4)	6.38 ± 0.04 (3)	112.3 ± 5.9 (3)	0.59 ± 0.13
Salmeterol	8.84 ± 0.09 (4)	59.7 ± 1.7 (4)	8.81 ± 0.08 (4)	12.0 ± 1.4 (4)	0.53 ± 0.15
Salbutamol	7.25 ± 0.04 (4)	67.7 ± 1.3 (4)	6.21 ± 0.09 (3)	19.1 ± 3.8 (3)	1.42 ± 0.19
Fenoterol	7.98 ± 0.05 (4)	77.0 ± 0.4 (4)	7.43 ± 0.08 (4)	85.0 ± 2.5 (4)	0.32 ± 0.11
Tulobuterol	7.14 ± 0.04 (4)	33.6 ± 0.6 (4)	ND	ND	ND
Alprenolol	8.06 ± 0.48 (4)	7.2 ± 0.5 (4)	ND	ND	ND

a In this case, the *E*_max_ for each pathway is expressed as the % maximum response of the pathway balanced ligand formoterol (set to 100%). In all cases, log bias factors (mG_s_/Arr2) were calculated in relation to the reference compound formoterol. Bias factors were calculated using the following equation: ΔΔlog­(*E*_max_/EC_50_). Data shown are from 3 or more experiments. All values are mean ± SEM from the indicated number of experiments shown in brackets.

To further validate our findings and capture the temporal dynamics of recruitment, we calculated the area under the curve (AUC) for each ligand’s mG_s_ and β-arrestin2 response profiles. These data are presented in Supporting Information Table S2. Notably, AUC-derived bias values were strongly correlated with those derived from peak responses (*R* ^2^ = 0.98), consistent with the fact that all ligands exhibited similar rise and decay kinetics (see Supporting Information Figure S2). This suggests that differences between ligands are primarily driven by amplitude rather than temporal features. These results reinforce our use of peak recruitment responses in the main analysis and demonstrate the temporal consistency of signaling across the conditions.

### Bias Signaling Plots Reveal Functional Selectivity

These apparent differences in bias are more easily observed by replotting the responses to these two pathways against each other at equimolar test concentrations. The four SABAs (see Figure 3 A–D) show very different profiles, with isoprenaline and isoetharine being the most similar. There is a clear bias toward mG_s_ recruitment at the lower concentrations tested (reflecting the potency differences at these two pathways). However, at higher concentrations, isoprenaline appears to recruit relatively higher levels of arrestin compared to the reference ligand formoterol. On the other hand, salbutamol shows relatively lower levels of mG_s_ recruitment and relatively minor levels of arrestin recruitment and only in the higher concentration range. Fenoterol is very weakly biased toward mG_s_ recruitment. In contrast, formoterol is very much balanced, while salmeterol, the other LABA tested, shows weaker recruitment of both mG_s_ and β-arrestin2 (Figure 3E and F). Plotting projected receptor occupancy over the same concentration ranges reveals an important feature of partial agonists such as tulobuterol, namely, that they tend to occupy the same number of receptors as full agonists but yet fail to produce a full maximal effect, in this case recruitment of mGs or β-arrestin2 protein (Figure 3G and H). The binding values used to construct receptor occupancy curves are detailed in Supporting Information Table S1.

**Figure 3:**
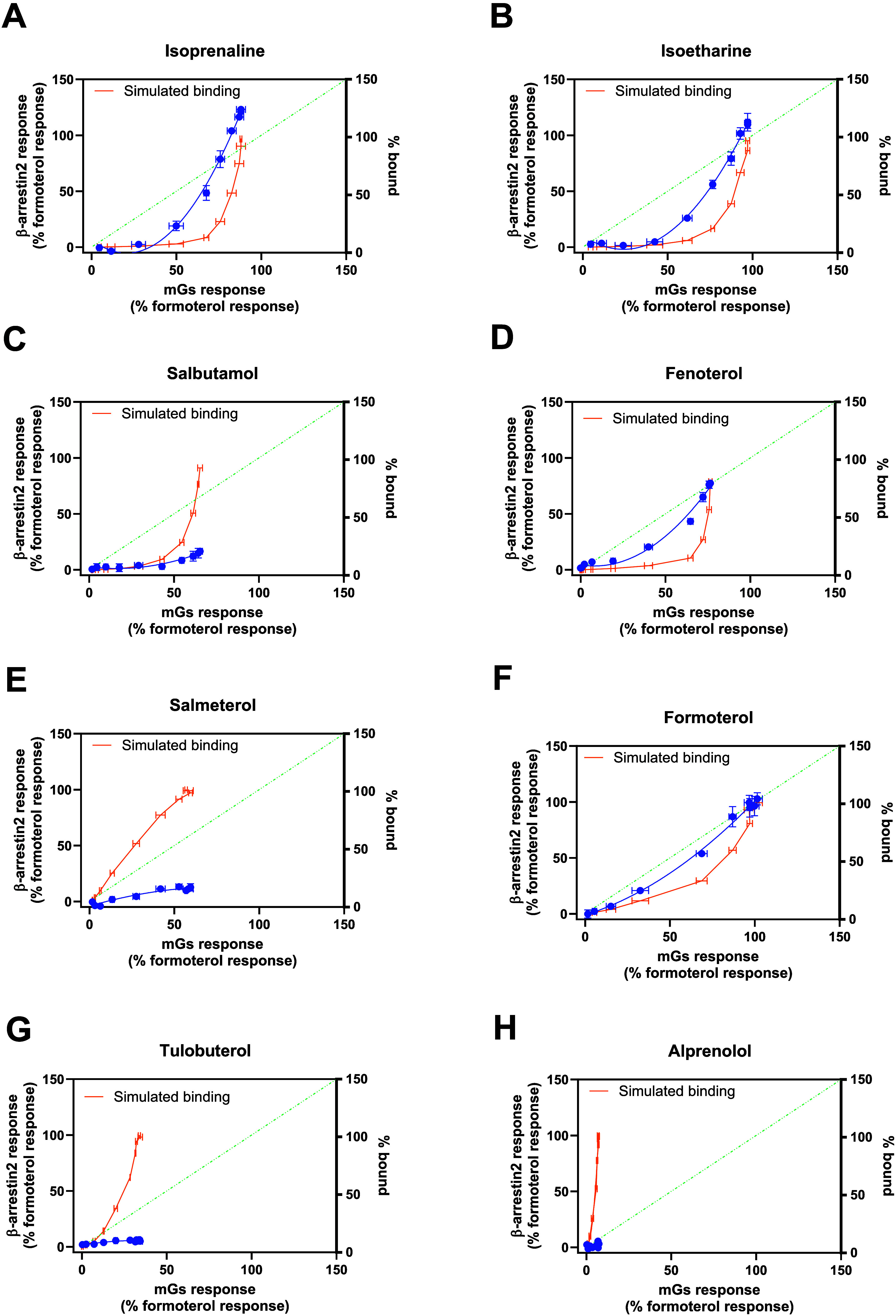
Qualitative bias plots for BRET-based β_2_AR mG_s_ and β-arrestin2 recruitment assay responses at equimolar concentrations. β_2_AR mG_s_ (*x*-axis) and β-arrestin2 responses (left *y*-axis) for the β_2_AR agonists (A) isoprenaline, (B) isoetharine, (C) salbutamol, (D) fenoterol, (E) formoterol, (F) salmeterol, (G) tulobuterol, and (H) alprenolol plotted at equimolar concentrations. The relative binding of each agonist to the β_2_AR is plotted (right *y*-axis) as a function of the mG_s_ response (*x*-axis), shown in orange (a connecting line is drawn between points), using p*K*_i_ values reported in the literature. The BRET ratios obtained were normalized using the pathway balanced ligand formoterol as the reference agonist (100% response). Data shown are the mean ± SEM from 3 or more experiments, with mG_s_ and β-arrestin2 responses to each agonist conducted on the same plate. A centered second-order polynomial fit of these response values in the respective assays is shown. *Whole cell binding data values are averages or single determinations taken from Baker^29,30^ and Onaran et al.^31^

### Calculation of Bias Factors

To rank the compounds based on pathway selectivity, ligand bias factors were calculated as a function of G_s_ and β-arrestin2 responses for the SABA/LABA under study and are represented as log bias factors (mG_s_/Arr2) in the bar chart (see Table 1 and Figure 4A). These results are also shown as bias factors (linear scale), along with the year each SABA/LABA was introduced to the market (Figure 4B). These results show that bias toward mG_s_ for both classes of agonist increases in the same rank order that the molecules were introduced to the market. The only exception is fenoterol, which was introduced last, but its bias factor is much weaker than salbutamol.

**Figure 4:**
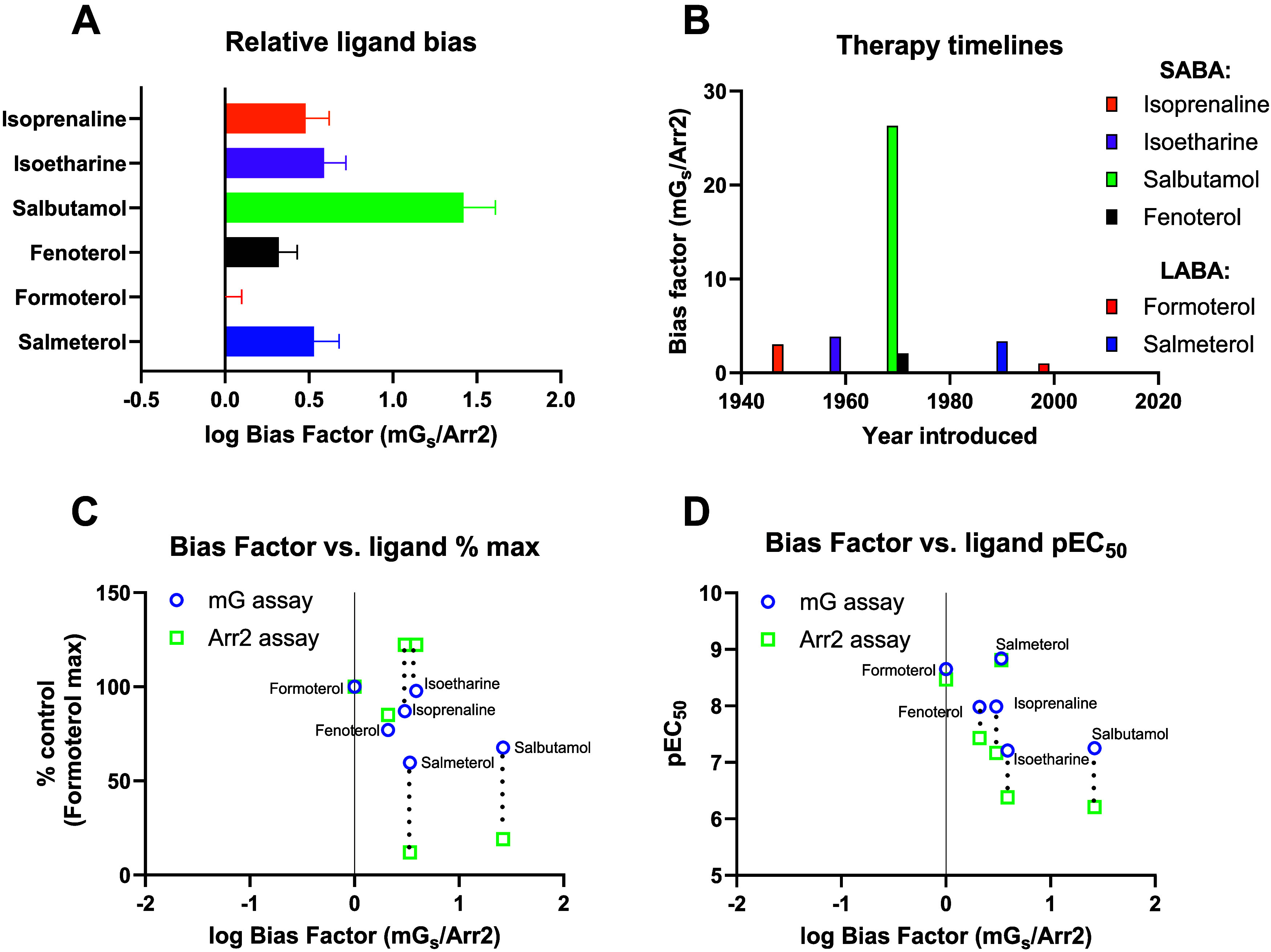
BRET-based β_2_AR mG_s_ and β-arrestin2 recruitment assay-derived ligand bias factors. Log bias factors were calculated as ΔΔlog­(*E*_max_/EC_50_), where pathway 1 is G protein (mG_s_)-dependent recruitment, and pathway 2 is β-arrestin2 recruitment. (A) mG_s_ and β-arrestin2 derived ligand bias factors (mG_s_/Arr2) and (B) a timeline for the introduction of clinically used SABAs and LABAs in relation to their bias factors (linear scale). The reference ligand chosen for the bias calculation was the pathway balanced ligand formoterol. Log bias factors are plotted as a function of G_s_ and β-arrestin2 (C) response and (D) potency. In all cases, log bias factors were calculated in relation to the reference compound formoterol. Bias factors were calculated by taking the antilog of the ΔΔlog­(*E*_max_/EC_50_). Data shown are from 3 or more experiments.

These same log bias factors are plotted as a function of G_s_ and β-arrestin2 response (*E*_max_) and potency (pEC_50_)—see Figure C and D. These plots highlight the changes in drug potency and *E*_max_ observed for each ligand at each pathway and help to rationalize the contribution of potency and *E*_max_ in the overall calculation of bias. In the case of tulobuterol, since an EC_50_ cannot be estimated for the β-arrestin2 pathway, a full quantitative bias factor calculation was not possible.

### Influence of Ligand Bias on β-Arrestin Recruitment Highlights the Superiority of Salbutamol

BRET-based β_2_AR mG_s_ and β-arrestin2 recruitment assay responses at three different ligand concentrations, specifically 1*, 3*, and 10* [EC_50_] in the mG_s_ assay, are plotted as a function of time. These plots show the clear effects of pathway bias on the relative levels of SABA-induced G_s_ protein and β-arrestin2 recruitment—see Figure 5A–L. From these plots, we can see that salbutamol shows only very modest increases in β-arrestin2 recruitment as its concentration is increased, whereas the other agents lose their apparent bias as their concentrations are increased.

**Figure 5:**
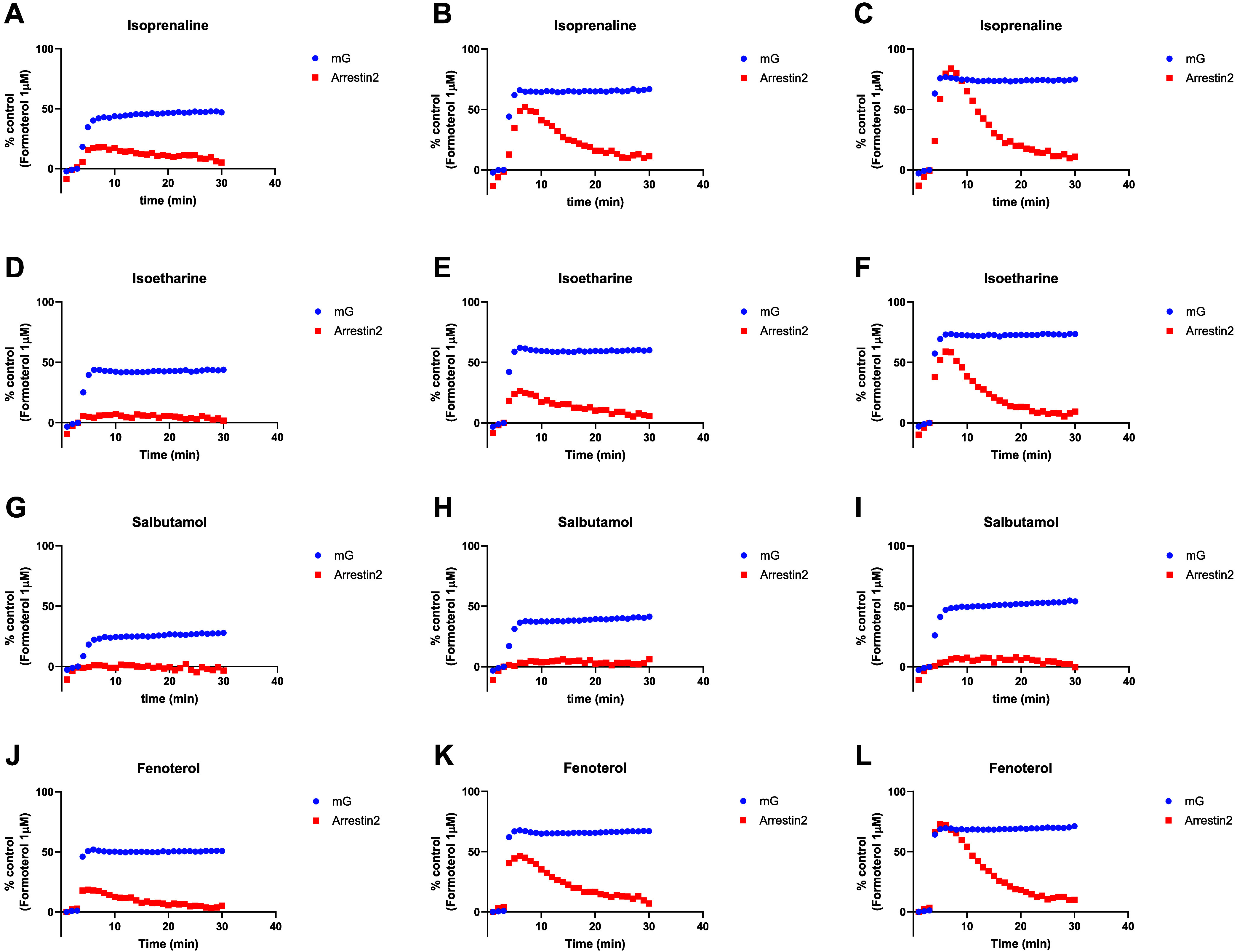
Kinetics of BRET-based β_2_AR mG_s_ and β-arrestin2 kinetic recruitment assay responses at different concentrations of SABAs. β_2_AR mG_s_ and β-arrestin2 responses at ligand [EC_50_] were determined in the mG_s_ assay. Individual β_2_AR mG_s_ and β-arrestin2 kinetic response curves are shown for: **(i)** isoprenaline at (A) 1*, (B) 3*, and (C) 10* its own [EC_50_] in the mG_s_ assay. **(ii)** isoetharine at (D) 1*, (E) 3*, and (F) 10* its own [EC_50_] in the mG_s_ assay. **(iii)** salbutamol at (G) 1*, (H) 3*, and **(I)** 10* its own [EC_50_] in the mG_s_ assay. **(iv)** fenoterol at **(J)** 1*, **(K)** 3*, and **(L)** 10* its own [EC_50_] in the mG_s_ assay. Data shown are representative of 3 experiments, with mG_s_ and β-arrestin2 responses to each agonist conducted on the same plate.

Similarly, β_2_AR mG_s_ and β-arrestin2 recruitment plots produced for two of the LABAs studied are shown in Figure 6A–F. Here, the profiles of salmeterol and formoterol are very different, with salmeterol recruiting very low levels of arrestin as its concentration is increased.

**Figure 6:**
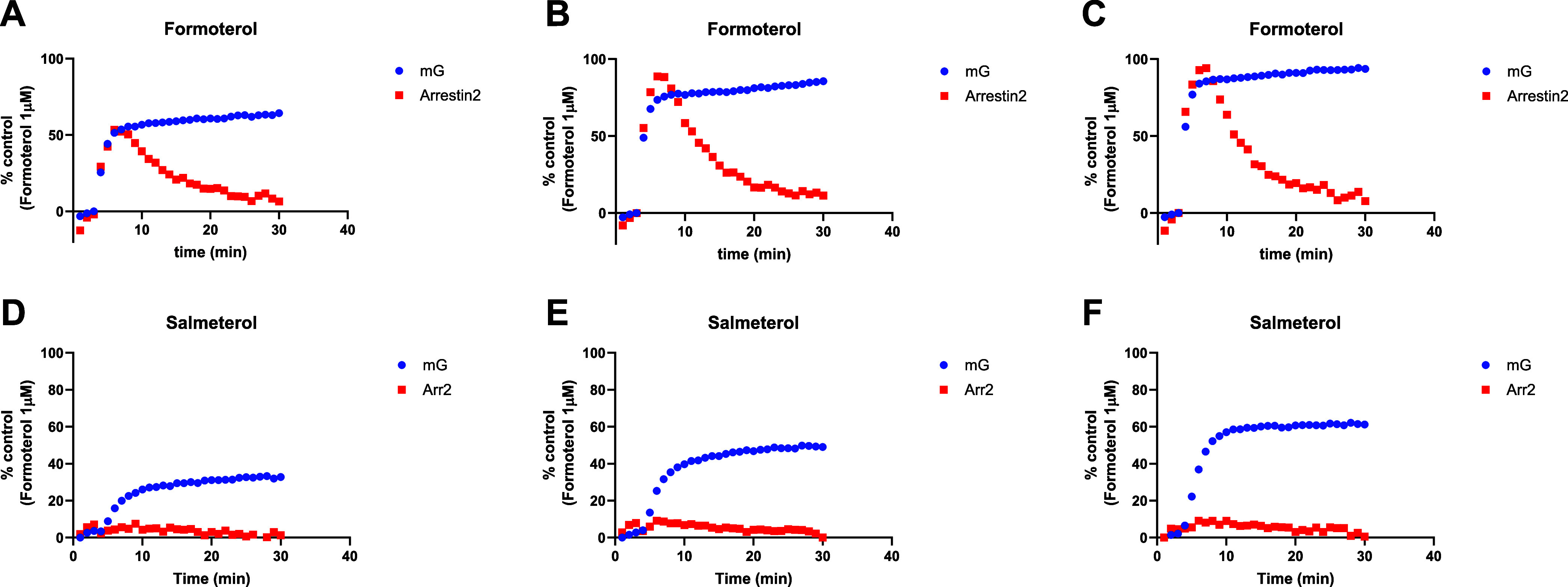
Kinetics of BRET-based β_2_AR mG_s_ and β-arrestin2 recruitment assay responses at different concentrations of LABAs. β_2_AR mG_s_ and β-arrestin2 responses at ligand [EC_50_] determined in the mG_s_ assay. Individual β_2_AR mG_s_ and β-arrestin2 kinetic response curves are shown for: **(i)** formoterol at (A) 1*, (B) 3*, and (C) 10* its own [EC_50_] in the mG_s_ assay. **(ii)** salmeterol at (D) 1*, (E) 3*, and (F) 10* its own [EC_50_] in the mG_s_ assay. Data shown are representative of 3 experiments, with mG_s_ and β-arrestin2 responses to each agonist conducted on the same plate.

### SABAs Dissociate Fast from Both the mG_s_ and β-Arrestin Receptor Complex

A series of separate experiments were performed at room temperature to look at both mG_s_ protein and β-arrestin recruitment reversibility. BRET-based β_2_AR mG_s_ and β-arrestin2 recruitment assay agonist pEC_50_ and *E*_max_ responses obtained at room temperature are detailed in Supporting Information Table S3. BRET-based β_2_AR mG_s_ recruitment assay agonist [EC_80_] responses are shown plotted as a function of time (see Supporting Information Figure S3A–G). These plots also show the reversibility of agonist mG_s_ responses starting at the 20 min time point, following the addition of a high concentration of the β_2_-blocker and inverse agonist ICI118551 (10 μM). In the mG_s_ assay, all four SABA mG_s_ responses reversed relatively faster than the two LABAs tested, formoterol and salmeterol. Tulobuterol, the weak partial agonist, was also relatively fast to reverse. Also shown in this figure are the BRET-based β_2_AR β-arrestin2 recruitment agonist [EC_80_] responses plotted as a function of time (see Supporting Information Figure S3H–K). Similarly, these plots show the reversibility of β-arrestin2 agonist responses following the addition of a high concentration of ICI118551 (10 μM). As seen in the mG_s_ assay, the β-arrestin2 responses of the two SABAs, isoprenaline and isoetharine, reversed relatively faster than the LABA, formoterol. Again, fenoterol also reversed β-arrestin2 recruitment relatively quickly, although more slowly than the more G protein–biased ligands isoprenaline and isoetharine. The recruitment signal by the weak partial agonists, salbutamol, salmeterol, and tulobuterol, was too small to be reliably reversed (data not shown).

Overall, these data suggest that the residence time (1/*k*_off_) of the ligand bound to the receptor–mG_s_ complex plays no role in the size of the mG_s_ response (*E*_max_). Similarly, the apparent ligand residence time of the ligand bound to the receptor−β-arrestin2 complex does not appear to play a role in the absolute levels of arrestin recruited (see Figure 7A and B and Supporting Information Table S4). Ligand pEC_50_ values obtained in the mG_s_ assay do not appear to be in any way related to measured log bias factors (mG_s_/Arr2); in contrast, a good correlation between log bias factors and β-arrestin2 pEC_50_ values was observed (see Figure 7C and D). The apparent ligand residence time of the ligand bound to both the receptor–mG_s_ complex appears to play a role in dictating mG_s_ pEC_50_ values. Similarly, the receptor−β-arrestin2 complex appears to play a role in dictating β-arrestin2 pEC_50_ values (see Figure 7E and F). As with β-arrestin2 pEC_50_ values, the measured residence time of ligands bound to the receptor−β-arrestin2 complex appears to be important in determining the log bias measurements obtained for ligands, which produced a measurable β-arrestin2 response (see Figure 7G and H).

**Figure 7:**
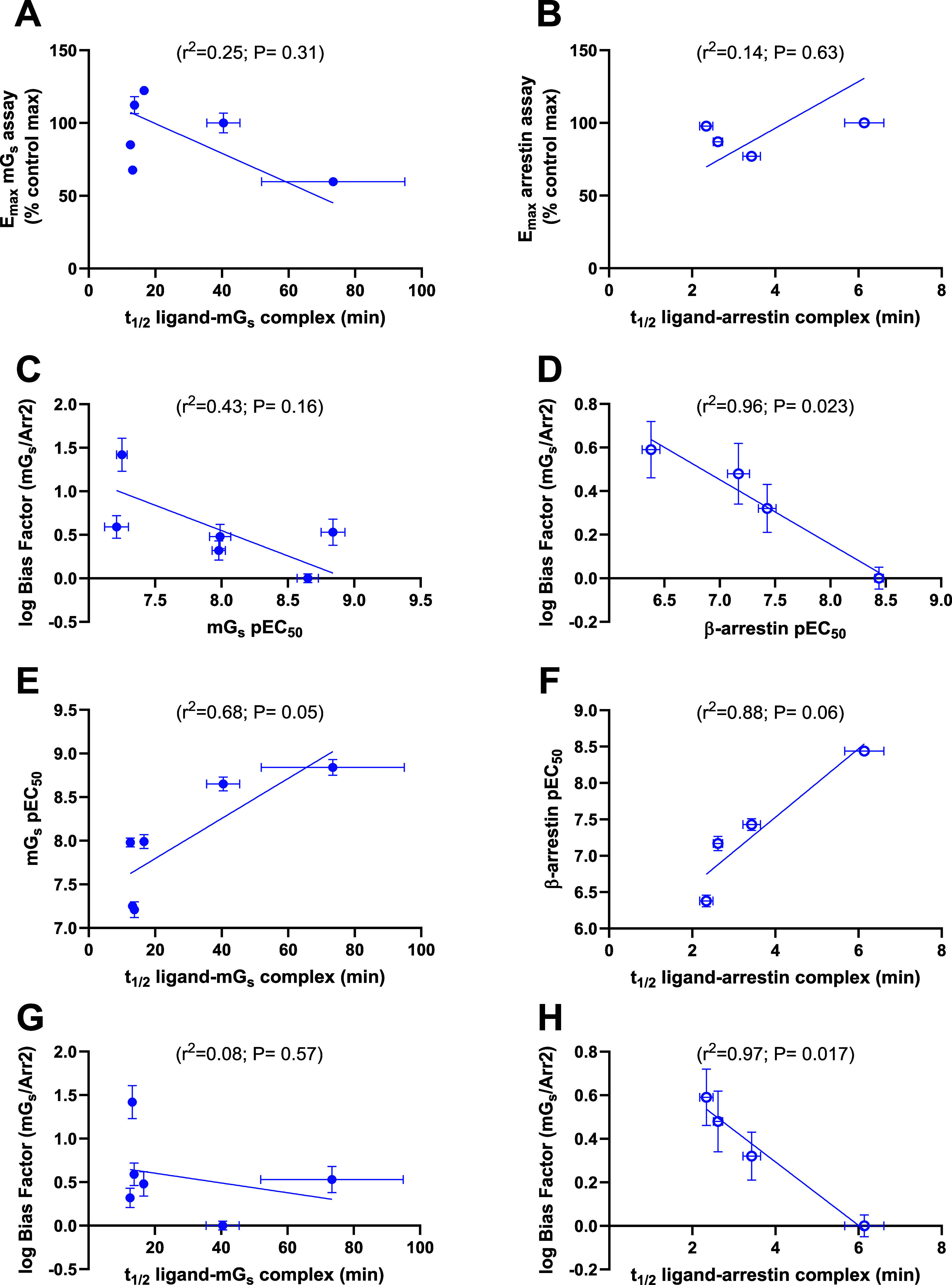
Ligand *t*_1/2_ values in relation to efficacy, potency, and bias. Correlation between ligand *t*_1/2_ values obtained by measuring the dissociation of ligands from receptor–mG_s_ and receptor−β-arrestin2 complexes and *E*_max_ values obtained in (A) mG_s_ and (B) β-arrestin2 recruitment assays. Correlation between ligands (C) mG_s_ pEC_50_ and (D) β-arrestin2 pEC_50_ values and log bias factors. Correlation between ligand *t*_1/2_ values obtained by measuring the dissociation of ligands from (E) receptor–mG_s_ and (F) receptor−β-arrestin2 complexes and ligand pEC_50_ values in the respective assays. Correlation between ligand *t*_1/2_ values obtained by measuring the dissociation of ligands from (G) receptor–mG_s_ and (H) receptor−β-arrestin2 complexes and the log bias factors. Ligand *t*_1/2_ values were obtained by applying a saturating concentration of the inverse agonist ICI118551 (10 μM) to mG_s_ and β-arrestin2 responses obtained at agonist [EC_80_]. All values are mean ± SEM from 3 or more experiments. In all cases, the reference ligand or control was formoterol. Dissociation *t*_1/2_ value (0.693/*k*_off_) is a surrogate for residence time (1/ *k*_off_).

## Discussion

While the deliberate design of biased ligands is a recent advancement, many approved drugs may unintentionally display functional bias due to optimization for therapeutic effects during development. We investigated this concept using the β_2_AR, a key clinical target in respiratory medicine, due to its well-characterized signaling pathways and historical role in biased ligand research. When the concept of the β_2_AR was first proposed,^32^ both structural information and a detailed understanding of the signaling molecules involved in cellular signal transduction were lacking. This limited knowledge meant that early research on respiratory drugs progressed slowly, relying primarily on pharmacological observations from organ bath experiments measuring airway smooth muscle relaxation. Given the technological limitations at the time, receptor desensitization was assessed by a rightward shift in potency or a decrease in maximal response (efficacy) in a second concentration–response curve, repeated on the same tissue after a washout period.

Advances in both structural biology and modern screening techniques have since dramatically improved our understanding of β_2_AR pharmacology and the process of desensitization. These developments have revealed that differential signal transduction is likely driven by subtle variations in drug binding poses, which in turn influence the stabilization of distinct receptor–effector conformations.^15,33^ To visualize these receptor conformations more effectively, we applied a straightforward BRET-based screening platform capable of distinguishing between β-arrestin-biased and G protein-biased ligands by assessing changes in maximal effector coupling (*E*_max_) and potency (EC_50_), allowing the calculation of ligand bias factors. In the current study, this system has detected the binding of fluorescently labeled mG_s_ very much in line with the reported efficacy of these ligands, determined using a cAMP assay.^29^ Importantly, our approach avoids signal amplification, reducing potential distortions in pathway bias measurements.^25^ This and other valuable experimental considerations are outlined in the review of Galandrin et al.^34^

At the start of this study, we theorized that the developments in existing β_2_AR ligands, including short-acting β_2_ agonists (SABAs) and long-acting β_2_ agonists (LABAs), would reflect their observed pharmacology, driven by improvements in preclinical and clinical efficacy, and as a consequence, signaling bias. The findings of this study reflect this hypothesis, demonstrating that improvements in the pharmacology of these compounds ultimately reflect their genesis and use in the clinic.

Isoprenaline was the first synthetic treatment introduced in the 1940s and originally marketed as a shorter-acting bronchodilator, but poor selectivity means it stimulates both β_1_ and β_2_AR at therapeutic concentrations, leading to tachycardia, its main unwanted side effect.^35^ Isoetharine followed and was introduced in the 1950s and originally developed to improve upon the nonselectivity of isoprenaline, being marketed as one of the first β_2_AR selective agonists.^36,37^ Early reports suggested that isoetharine exhibited β-arrestin-biased signaling.^38,39^ However, recent studies, including the current one, contradict this view, highlighting a shift in the understanding of its signaling bias as a molecule, which strongly favors the G_s_ signaling pathway.^40^ Clinical data for isoetharine tend to support an apparent G protein bias, complementing its observed rapid onset of action and improved clinical performance.^41,42^

Isoetharine’s use declined as more effective and longer-lasting noncatecholamine-based β_2_AR selective agonists were developed.^43^ One such agent, salbutamol (or albuterol), was introduced in 1969 ^37^ and remains the standard treatment for acute asthma, being one of the most prescribed drug therapies in the USA and the world (https://clincalc.com/DrugStats/Drugs/Albuterol). Importantly, the partial agonist effect of salbutamol, observed in the current study in both the mG_s_ recruitment and β-arrestin assays, does not appear to translate into reduced effectiveness in lung function control, compared to the fuller agonist isoetharine.^42,44^ One mechanism for this observation could be reduced receptor desensitization and internalization observed with partial β_2_AR agonists such as salbutamol compared to fuller agonists, such as adrenaline, or alternatively, the benefit of a high level of β_2_AR and/or improved G_s_ coupling in the lung.^45,46^ Indeed, regular use of the higher efficacy but weakly biased fenoterol was associated with worsening asthma symptoms and tolerance, which may partly explain the increased mortality associated with use of this agent, culminating in its eventual withdrawal in certain countries.^47^

Desensitization of the β_2_AR due to prolonged stimulation from agonists can blunt both the bronchodilatory and the antibronchoconstrictor effects of β_2_ agonists in asthma. Tolerance to regular formoterol use has been demonstrated in challenge rescue models, where airway constriction induced by methacholine (an M_3_ agonist) is followed by high-dose salbutamol to assess reduced bronchodilation in response to formoterol.^48,49^ Tolerance to the broncho-protective and broncho-dilative effects of inhaled LABAs occurs remarkably rapidly, after only one or two doses,^48,50,51^ whereas tolerance to salbutamol is observed less frequently^52,53^ or not at all.^54^ Other studies have demonstrated a reduction in its bronchodilator efficacy during acute bronchoconstriction with daily inhaler use,^55^ although frequent use (>4× in 24 h, and more than 2 days per week) is not recommended. Tulobuterol, administered via a transdermal patch, is unique in that it does not recruit significant levels of β-arrestin2, nor does its repeated administration lead to significant tolerance, highlighting the benefit of complete G protein bias.^56^

One study comparing continuous stimulation of the β_2_AR in human primary bronchial smooth muscle cells with β_2_AR agonists clearly demonstrated that a short stimulation period with the high efficacy ligand isoprenaline led to less desensitization than longer stimulations with the LABAs formoterol and salmeterol.^57^ It is not unreasonable to assume that for short-acting β_2_ agonists such as salbutamol, which are more readily eliminated from the body, the receptor has more time to resensitize between doses, allowing restoration of β_2_AR responsiveness, whereas, formoterol, due to its prolonged action, provides little opportunity for receptor resensitization, leading to progressive loss of efficacy with regular use and tolerance. The less frequent administration of agents such as formoterol (once daily) and their combination with steroids has been suggested as a mechanism for diminished tolerability, due to steroid-induced β_2_AR upregulation.^58,59^

Tachyphylaxis and internalized receptor number has been linked to receptor target coverage, so it is entirely possible that the fast dissociation profile of salbutamol observed in this study contributes to its improved efficacy on repeat dosing and even ligand bias^60–64^ — see Figure 8.

**Figure 8:**
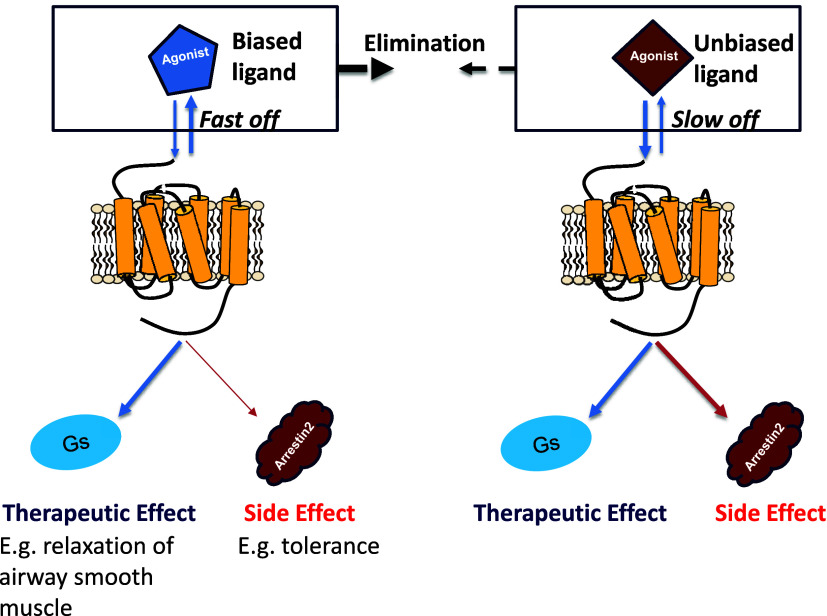
Rapid ligand-β_2_ adrenoceptor target reversibility, the result of both rapid drug elimination from the body and faster ligand dissociation from the receptor−β-arrestin2 complex, leads to overall reduced β-arrestin2 recruitment and potentially a reduction in tolerance to repeat dosing.

The World Health Organization (WHO) ranks salbutamol as one of the most effective and safest medicines essential to healthcare systems.^65^ In the current study, this agent shows the greatest degree of signaling bias toward the G protein of all of the agents tested, including the two LABAs salmeterol and formoterol.

The main advantage of the BRET-based mG_s_ and β-arrestin2 systems used in this study is their high sensitivity and reproducibility, partly due to the generation of stable cell lines coexpressing the receptor and fluorescent effector proteins. However, this study is not without limitations, and it must be recognized that more physiologically relevant signaling systems have been created to assess signaling bias. The most developed of these is the TRUPATH system, which enables a comprehensive profiling of GPCR signaling bias by reconstituting the full heterotrimeric G protein complex in cells.^66^ Much like the mG system described herein, TRUPATH provides a useful platform for assessing pathway-specific signaling in a high-throughput manner.

Nonetheless, despite these advances in assay design, the interpretation of signaling bias remains complex. While the clinical reliability of salbutamol and other β_2_AR ligands may be influenced by their signaling bias profiles, current evidence from the limited number of studies discussed remains preliminary and insufficient to establish a direct causal relationship between in vitro signaling preferences (e.g., Gs vs β-arrestin) and clinical outcomes. And although our previous work demonstrated a strong correlation between mG_s_ recruitment and GsCASE activation,^67^ mG protein recruitment in general is not necessarily equivalent to functional G protein activation or dissociation in all GPCR systems. Similarly, β-arrestin translocation alone does not confirm the downstream signaling activity. Further research is needed to clarify the role of signaling bias in desensitization, tolerance, and inflammation in asthma and COPD.

## Conclusions

This study used BRET-based technology to assess GPCR ligand efficacy at two different signaling pathways by tracking fluorescently labeled effector proteins. Based on the results presented here, we suggest that future drug discovery programs should consider nonamplified, real-time technologies to minimize readout bias, as these approaches allow for immediate detection of ligand–receptor interactions, reducing signal distortion and improving accuracy.^68^ A list of biosensors available for use in future drug discovery pipeline strategies is provided in the following reviews, which cover G protein biosensors and arrestin biosensors.^69,70^ Integrating such precise signaling assessments early in the drug discovery process offers significant potential to address safety and efficacy concerns upfront, ultimately reducing costly failures in later development phases and enhancing the likelihood of clinical success.

## Supplementary Material



## Notes

Supporting figures showing agonist time courses in the mG_s_ and arrestin2 assays and assay schematics. Tables summarizing literature whole cell binding affinity values, plus tables summarizing kinetic (AUC analysis) and room temperature equilibrium parameters from mG_s_ and β-arrestin2 recruitment assays, plus reversibility experiments (PDF)

## Abbreviations

GPCR: G protein-coupled receptor
β_2_AR: β_2_-adrenergic receptor
SABAs: short-acting β_2_AR agonists
LABAs: long-acting β_2_AR agonists
BRET: bioluminescence resonance energy transfer
mG_s_: mini-Gs protein
Arr2: β-arrestin2
