# Supplementary material for: Biased Signaling and Its Role in the Genesis of Short- and Long-Acting β2‑Adrenoceptor Agonists
Source: Biochemistry. 2025 Aug 7;64(16):3585–98. doi: 10.1021/acs.biochem.5c00148 (PMC12369021; doi:10.1021/acs.biochem.5c00148)
Supplement: Supplementary file 1 [file bi5c00148_si_001.pdf]

Supporting Information

Biased signaling and its role in the genesis of short and long acting β_2-adrenoceptor agonists

Ngan T.N. Phan[1, 2], Leire Borrega-Roman[1,2], Bradley L. Hoare[1,2], Clare R. Harwood[1,2], Natalie Geary[2], Wolfgang Guba[4], Yongqi Han[1,2], Vladimirs Zenko[1,2], Eline J. Koers[1,2], Arne C Rufer[4,*], Uwe Grether[4,*], Dmitry B. Veprintsev[1,2,3,*] and David A Sykes[1,2,3,*]

1. Division of Physiology, Pharmacology & Neuroscience, School of Life Sciences, University of Nottingham, Nottingham, NG7 2UH, UK

2. Centre of Membrane Proteins and Receptors (COMPARE), University of Nottingham, Midlands, NG7 2UH, UK

3. Z7 Biotech Limited, 1 Poplars Court, Lenton Lane, Nottingham, NG7 2RR, UK

4. Roche Pharma Research & Early Development, Therapeutic Modalities, Roche Innovation Center Basel, F. Hoffmann-La Roche Ltd., 4070 Basel, Switzerland

Corresponding author(s):

Dr David A. Sykes

School of Life Sciences,

Queen’s Medical Centre,

University of Nottingham,

Nottingham NG7 2UH

david.sykes@nottingham.ac.uk or

david.sykes@Z7bio.com

Prof Dmitry B. Veprintsev

School of Life Sciences,

Queen’s Medical Centre,

University of Nottingham,

Nottingham NG7 2UH

dmitry.veprintsev@nottingham.ac.uk or

dmitry.veprintsev@Z7bio.com

Dr Uwe Grether and Dr Arne Rufer

Roche Pharma Research & Early Development,

Roche Innovation Center Basel,

F. Hoffmann-La Roche Ltd.,

4070 Basel, Switzerland,

E-mail: uwe.grether@roche.com and arne.rufer@roche.com

Figure S1.

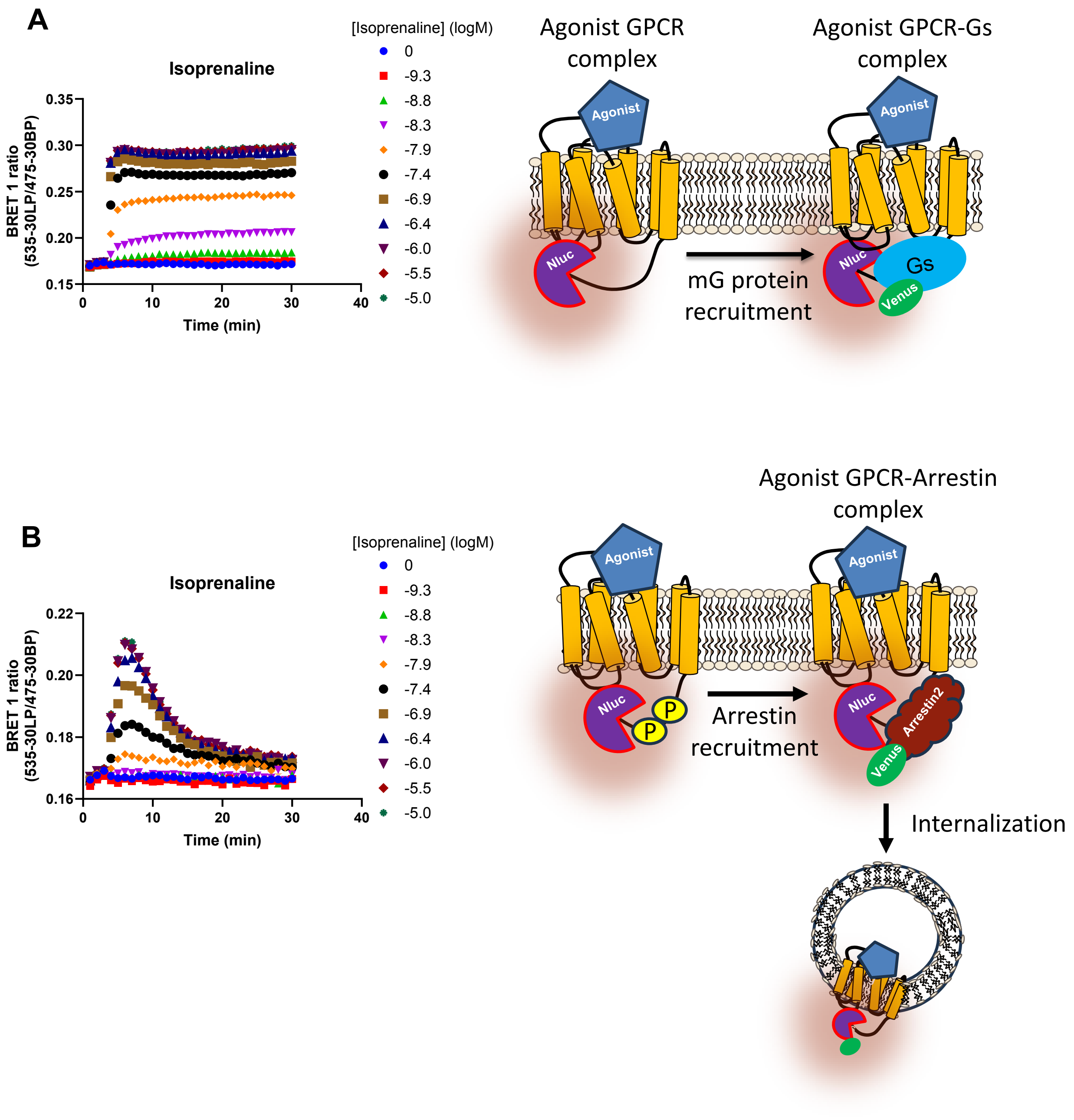

Figure S1. mG$_s$ and β-arrestin2 time course and assay schematics. Time course of isoprenaline induced **(A)** mG$_s$ and **(B)** β-arrestin2 recruitment in the mG$_s$ and β-arrestin2 binding assay. Data are typical traces from a single experiment.

Figure S2

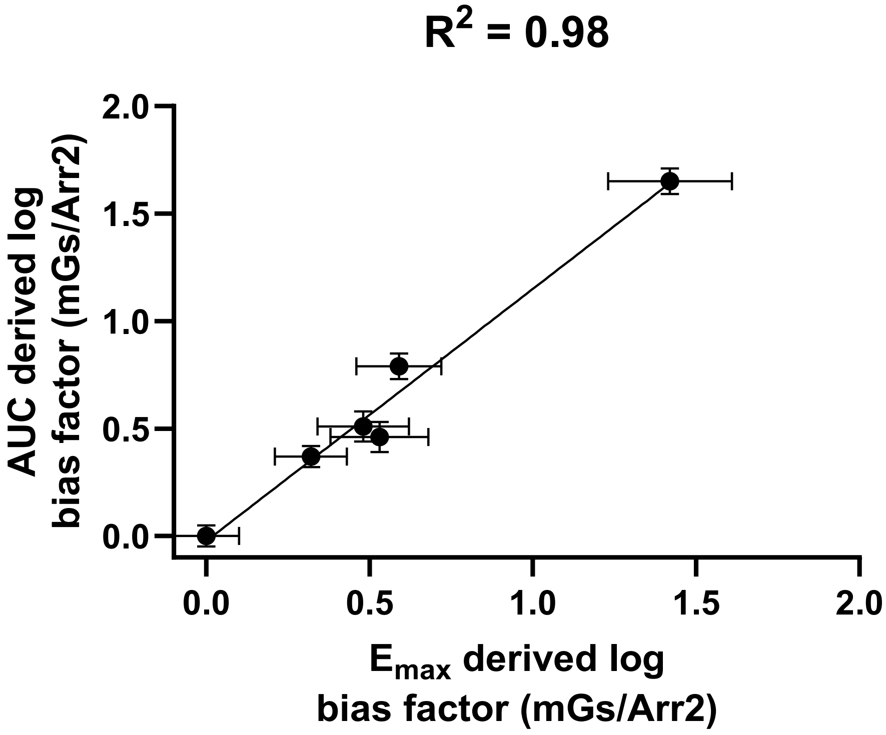

Figure S2. Correlation between maximal response and AUC analysis derived ligand bias factors. BRET-based β_2AR mG_s and β-arrestin2 recruitment assay maximal response derived ligand bias factors are plotted on the X-axis and AUC derived values are plotted on the Y-axis. Log bias factors were calculated as $\Delta\Delta\log(E_{max}/EC_{50})$ where pathway 1 is G-protein (mG_s) dependent recruitment and pathway 2 is β-arrestin2 recruitment. In all cases log bias factors were calculated in relation to the reference compound formoterol. Data shown are from a 3 or more experiments.

Figure S3

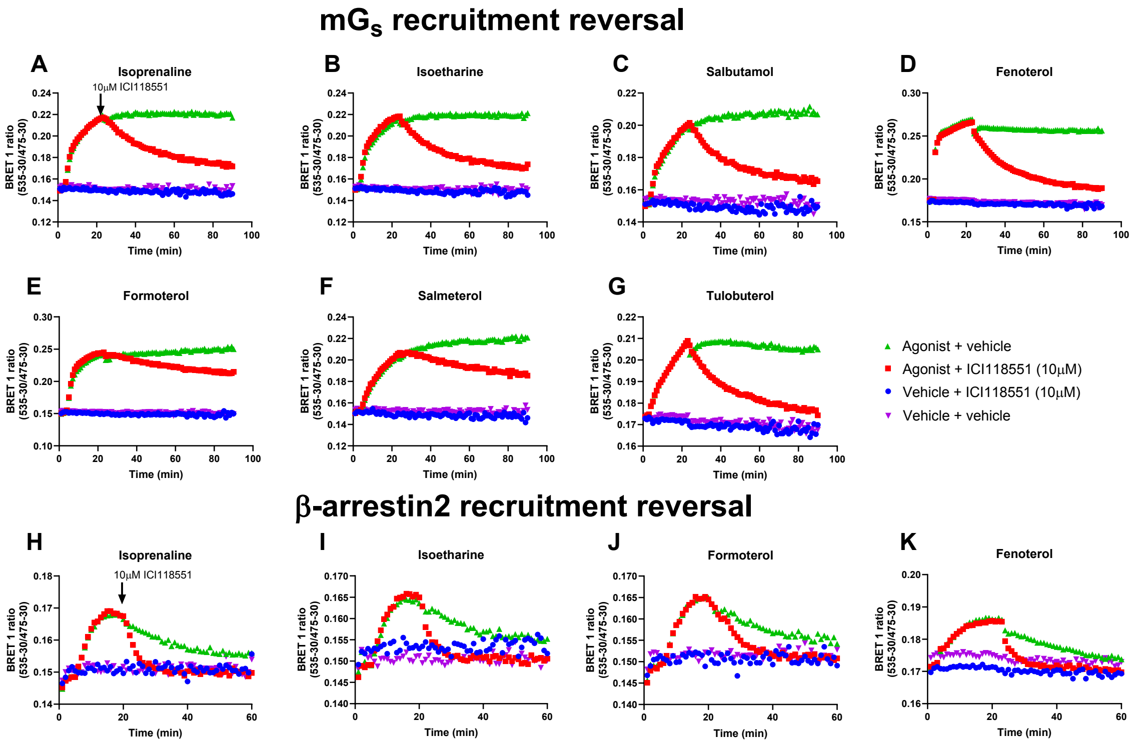

Figure S3. Reversibility of mG_s and β-arrestin2 recruitment. β_2AR mG_s responses (shown in green) to β_2AR agonists **(A)** isoprenaline, **(B)** isoetharine, **(C)** salbutamol, **(D)** fenoterol, **(E)** formoterol, **(F)** salmeterol and **(G)** tulobuterol applied at an [EC_{80}] plotted as a function of time. β-arrestin2 responses (shown in green) to β_2AR agonists **(H)** isoprenaline, **(I)** isoetharine, **(J)** formoterol, and **(K)** fenoterol applied at an [EC_{80}] plotted as a function of time. Recruitment reversal (shown in red) was initiated by the addition of a high concentration of the inverse agonist ICI118551 (10μM). Data are shown as the mean value from 5 independent observations.

Table S1.

**Compound**	**Literature values**	**Average pK_i**
**Formoterol**	*8.63, **7.6	8.12
**Isoprenaline**	*6.64, **6.3	6.47
**Isoetharine**	**5.8	5.8
**Salmeterol**	*9.26, **8.8	9.03
**Salbutamol**	*6.12, **5.9	6.01
**Fenoterol**	*7.03, **6.1	6.57
**Tulobuterol**	*6.83, **6.6	6.72
**Alprenolol**	***9.04	9.04

Table S1. Whole cell binding affinity values. Binding values were taken from *Baker 2010 [1], **Onaran et al., 2017 [2] and **Baker 2010 [3].

Table S2.

β_2AR					
**Compound**	**mG_s**		**β-arrestin2**		**Log Bias Factor (mG_s/Arr2)**
	**pEC_{50}**	**E_{max}**	**pEC_{50}**	**E_{max}**	
**Formoterol**	8.66 ± 0.16 (4)	100 ± 0.7 (4)	8.73± 0.15 (3)	100 ± 2.16 (3)	0 ± 0.05
**Isoprenaline**	7.95 ± 0.07 (8)	92.5 ± 3.2 (8)	7.41 ± 0.08 (7)	118.7 ± 11.8 (7)	0.51 ± 0.07
**Isoetharine**	7.42 ± 0.07 (4)	93.9 ± 1.7 (4)	6.65 ± 0.09 (3)	102.7 ± 4.37 (3)	0.79 ± 0.06
**Salmeterol**	8.69 ± 0.09 (4)	67.2 ± 1.8 (4)	9.17 ± 0.33 (4)	18.3 ± 2.1 (4)	0.46 ± 0.07
**Salbutamol**	7.21 ± 0.04 (4)	65.0 ± 2.0 (4)	6.08 ± 0.24 (3)	32.0 ± 8.4 (3)	1.65 ± 0.06
**Fenoterol**	7.99 ± 0.04 (4)	76.7 ± 0.8 (4)	7.70 ± 0.04 (4)	93.6 ± 8.1 (4)	0.37 ± 0.05
**Tulobuterol**	7.11 ± 0.04 (4)	32.8 ± 0.4 (4)	ND	ND	ND
**Alprenolol**	8.79 ± 0.13 (4)	7.6 ± 0.5 (4)	ND	ND	ND

Table S2. BRET-based mG_s and β-arrestin2 recruitment maximal effect (E_{max}) and potency (pEC_{50}) data derived from AUC analysis plus the resulting bias factors. In this case the E_{max} for each pathway is expressed as the % maximum response of the pathway balanced ligand formoterol (set to 100%). In all cases log bias factors (mG_s/Arr2) were calculated in relation to the reference compound formoterol. Bias factors were calculated using the following equation $\Delta\Delta\log(E_{max}/EC_{50})$. Data shown are from a 3 or more experiments. All values are mean ± SEM from the indicated number of experiments shown in brackets.

Table S3.

β_2AR				
**Compound**	**mG_s**		**β-arrestin2**	
	**pEC_{50}**	**E_{max}**	**pEC_{50}**	**E_{max}**
**Formoterol**	8.76 ± 0.13	100 ± 0.7	8.78 ± 0.11	100 ± 6.4
**Isoprenaline**	8.43 ± 0.14	86.3 ± 2.7	7.48 ± 0.18	114.8 ± 4.1
**Isoetharine**	7.87 ± 0.10	101.3 ± 2.8	6.68 ± 0.13	116.2 ± 1.7
**Salmeterol**	8.58 ± 0.12	78.9 ± 1.8	8.91 ± 0.17	11.8 ± 1.1
**Salbutamol**	7.25 ± 0.04	78.1 ± 1.1	6.45 ± 0.17	22.1 ± 1.1
**Fenoterol**	8.10 ± 0.01	73.9 ± 2.1	7.53 ± 0.05	83.8 ± 7.1
**Tulobuterol**	7.08 ± 0.10	41.9 ± 1.4	7.25 ± 0.72	8.8 ± 1.0

Table S3. BRET-based mG_s and β-arrestin2 recruitment maximal effect (E_{max}) and potency (pEC_{50}) data obtained at room temperature. In this case the E_{max} for each pathway is expressed as the % maximum response of the pathway balanced ligand formoterol (set to 100%). Data are mean ± SEM from 3 independent observations.

Table S4.

β_2AR				
**Compound**	**mG_s**		**β-arrestin2**	
	**k_{off} (min^{-1})**	**½ (min)**	**k_{off} (min^{-1})**	**½ (min)**
**Formoterol**	0.018 ± 0.002	40.5 ± 5.0	0.12 ± 0.01	6.14 ± 0.47
**Isoprenaline**	0.042 ± 0.001	16.6 ± 0.5	0.27 ± 0.01	2.62 ± 0.11
**Isoetharine**	0.051 ± 0.002	13.7 ± 0.5	0.30 ± 0.02	2.34 ± 0.16
**Salmeterol**	0.012 ± 0.002	73.4 ± 21.5	ND	ND
**Salbutamol**	0.053 ± 0.002	13.2 ± 0.4	ND	ND
**Fenoterol**	0.055 ±0.001	12.5 ± 0.3	0.20 ± 0.01	3.43 ± 0.21
**Tulobuterol**	0.049 ± 0.002	14.2 ± 0.6	ND	ND

Table S4. BRET-based mG_s and β-arrestin2 ligand dissociation and $t_{1/2}$ values obtained by measuring the dissociation of ligands from receptor-mG_s and receptor-β-arrestin2 complexes with a saturating concentration of the inverse agonist ICI118551 (10 μM). Data are mean ± SEM from 5 independent observations.

Estimation of pathway error:

Error on the pathway bias was calculated using a general procedure for error calculation via Gaussian propagation law for correlated data, valid when errors are small and normally distributed:

$$SE_f = \sqrt{\sum_i^n \left(\frac{\partial f}{\partial x_i} SE_{x_i}\right)^2 + 2\sum_{i<j}\left(\frac{\partial f}{\partial x_i}\frac{\partial f}{\partial x_j} Cov(x_i,x_j)\right)} \qquad \text{Eq. 1}$$

Where $f(x_1,x_2,\dots,x_n)$ is a function of propagated variables, SE_{x_i} is the Standard Error of each propagated variable and estimates the standard deviation of the statistical distribution, $\partial f/\partial x_i$ is the partial derivative of function with respect to its propagated variable and where covariance terms $Cov(x_i,x_j)$ account for dependencies between the two variables (calculated numerically). Note: A Gaussian formula can be used with SE as it is an estimate of Standard Deviation of the statistical distribution. Standard Error of the Mean, SEM, is a special case of SE used when the propagated variables represent sample means:

$$SEM = \frac{\sigma}{\sqrt{n}} \qquad \text{Eq. 2}$$

where σ is the Standard Deviation, and n is the number of experiments performed. SEM is used for the analysis since it accounts for difference in distribution sample size (number of experiments per pathway). SEM can be thought of as normalised Standard Deviation, whereas the Standard Deviation σ quantifies the scatter of the data. Using Equation 5, a single SEM value was calculated for each distribution of E_{max} and EC_{50} over all experiments for both test (A, Ligand 1) and reference (B, Ligand 2) ligands, and for both pathways. SEM accounts for differences in sample sizes across distributions, making it a normalized measure of variability. By transforming Equation 2, using logarithm properties, a simplified form of the agonist activity difference for the two pathways was generated:

$$\Delta\log\left(\frac{E_{max}}{EC_{50}}\right) = \log\left(E_{max}^{B}\right) - \log\left(\mathrm{EC}_{50}^{B}\right) - \log\left(E_{max}^{A}\right) + \log\left(\mathrm{EC}_{50}^{A}\right)$$

$$\Rightarrow$$

$$\Delta\log\left(\frac{E_{max}}{EC_{50}}\right)_{P1} = \log\left(E_{max}^{B,P1}\right) - \log\left(\mathrm{EC}_{50}^{B,P1}\right) - \log\left(E_{max}^{A,P1}\right) + \log\left(\mathrm{EC}_{50}^{A,P1}\right) \qquad \text{Eq. 3}$$

$$\Delta\log\left(\frac{E_{max}}{EC_{50}}\right)_{P2} = \log\left(E_{max}^{B,P2}\right) - \log\left(\mathrm{EC}_{50}^{B,P2}\right) - \log\left(E_{max}^{A,P2}\right) + \log\left(\mathrm{EC}_{50}^{A,P2}\right)$$

Where E_{max} and EC_{50} values under the log function represent the mean values for each ligand and pathway, averaged over the number of experiments performed. While individual measurements of E_{max} and EC_{50} had reported errors, the Standard Error of the Mean (SEM) was preferred for estimating uncertainty. SEM provides a measure of how precisely the mean represents the true population value, accounting for variations across experiments. To quantify the error in the activity difference term, the SEM values for E_{max} and EC_{50} were then propagated using Equation 1. The activity

difference term was treated as a function of E_{max} and EC_{50} for both ligands. By computing the partial derivatives, the SEM of the agonist activity difference term was determined, taking the following form:

$$\mathrm{SEM}^2_{\Delta\log\left(\frac{E_{max}}{EC_{50}}\right)_{P1/2}} = \left(\frac{SEM_{E_{max}^{B,P1/2}}}{E_{max}^{B,P1/2}\ln(10)}\right)^2 + \left(\frac{SEM_{EC_{50}^{B,P1/2}}}{EC_{50}^{B,P1/2}\ln(10)}\right)^2 + \left(\frac{SEM_{E_{max}^{A,P1/2}}}{E_{max}^{A,P1/2}\ln(10)}\right)^2 + \left(\frac{SEM_{EC_{50}^{A,P1/2}}}{EC_{50}^{A,P1/2}\ln(10)}\right)^2 + 2\left(\frac{SEM_{E_{max}^{A,P1/2}}}{E_{max}^{A,P1/2}\ln(10)}\frac{SEM_{EC_{50}^{A,P1/2}}}{EC_{50}^{A,P1/2}\ln(10)}Cov\left(E_{max}^{A,P1/2}, EC_{50}^{A,P1/2}\right)\right) + 2\left(\frac{SEM\ [illegible]}{E_{max}^{B,P1/\ [illegible]}}\ [illegible]\right. \qquad \text{Eq. 4}$$

In Equation 7, cross-ligand covariance terms were not included because each ligands potency and efficacy were measured separately and independently. The covariance terms were instead calculated numerically from the distribution of E_{max} and EC_{50} data. To estimate the error in the pathway bias, the uncertainties of the $\Delta\log(E_{max}/EC_{50})$ terms were propagated into Equation 3. Since these $\Delta\log(E_{max}/EC_{50})$ terms had already been computed along with their corresponding uncertainties, they could be treated as direct measurables. As a result, there was no need to apply logarithmic error propagation for E_{max} and EC_{50} individually. Thus, a simplified Gaussian error propagation formula based on the standard addition/subtraction rule was applied:

$$SE_f = \sqrt{\sum_i^n \left(\frac{\partial f}{\partial x_i} SE_{x_i}\right)^2} \qquad \text{Eq. 5}$$

Taking SE_f as $SEM_{\Delta\Delta\log\left(\frac{E_{max}}{EC_{50}}\right)}$, $\Delta\Delta\log(E_{max}/EC_{50})$ as function f, and $\Delta\log\left(\frac{E_{max}}{EC_{50}}\right)_{P1,\,P2}$ as variables x_1, x_2 we were allowed to treat the pathway bias as a difference of two measurable variables:

$$\mathrm{f}(\mathrm{x}_1,\mathrm{x}_2) = \Delta\Delta\log\left(\frac{E_{max}}{EC_{50}}\right) = x_1 - x_2 \qquad \text{Eq. 6}$$

Hence, the partial derivatives are simply:

$$\frac{\partial f}{\partial x_1} = 1 \quad ; \quad \frac{\partial f}{\partial x_2} = -1 \qquad \text{Eq. 7}$$

This simplification reduces the Gaussian error propagation formula to its final form for the error on the pathway bias:

$$SEM_{\Delta\Delta\log\left(\frac{E_{max}}{EC_{50}}\right)} = \sqrt{\mathrm{SEM}^2_{\Delta\log\left(\frac{E_{max}}{EC_{50}}\right)_{P1}} + \mathrm{SEM}^2_{\Delta\log\left(\frac{E_{max}}{EC_{50}}\right)_{P2}}} \qquad \text{Eq. 8}$$

This aligns with the results of error propagation derivation outlined in van der Westhuizen *et al*. [4], which justifies the use of Gaussian propagation law.

Note: since covariance was already calculated when propagating errors on original E_{max} and EC_{50} measurements, it did not need to be included again when propagating errors from the activity difference terms onto the pathway bias.

References

1. Baker, J. G., The selectivity of beta-adrenoceptor agonists at human beta1-, beta2- and beta3-adrenoceptors. *Br J Pharmacol* **2010,** *160* (5), 1048-61.

2. Onaran, H. O.; Ambrosio, C.; Ugur, O.; Madaras Koncz, E.; Gro, M. C.; Vezzi, V.; Rajagopal, S.; Costa, T., Systematic errors in detecting biased agonism: Analysis of current methods and development of a new model-free approach. *Sci Rep* **2017,** *7*, 44247.

3. Baker, J. G., A full pharmacological analysis of the three turkey beta-adrenoceptors and comparison with the human beta-adrenoceptors. *PLoS One* **2010,** *5* (11), e15487.

4. van der Westhuizen, E. T.; Breton, B.; Christopoulos, A.; Bouvier, M., Quantification of ligand bias for clinically relevant beta2-adrenergic receptor ligands: implications for drug taxonomy. *Mol Pharmacol* **2014,** *85* (3), 492-509.
